# Effects of landscape anthropization on mosquito community composition and abundance

**DOI:** 10.1038/srep29002

**Published:** 2016-07-04

**Authors:** Martina Ferraguti, Josué Martínez-de la Puente, David Roiz, Santiago Ruiz, Ramón Soriguer, Jordi Figuerola

**Affiliations:** 1 Estación Biológica de Doñana (EBD-CSIC), Seville, Spain; 2 CIBER Epidemiología y Salud Pública (CIBERESP), Spain; 3 Diputación de Huelva, Área de Medio Ambiente, Servicio de Control de Mosquitos, Huelva, Spain

## Abstract

Anthropogenic landscape transformation has an important effect on vector-borne pathogen transmission. However, the effects of urbanization on mosquito communities are still only poorly known. Here, we evaluate how land-use characteristics are related to the abundance and community composition of mosquitoes in an area with endemic circulation of numerous mosquito-borne pathogens. We collected 340 829 female mosquitoes belonging to 13 species at 45 localities spatially grouped in 15 *trios* formed by 1 urban, 1 rural and 1 natural area. Mosquito abundance and species richness were greater in natural and rural areas than in urban areas. Environmental factors including land use, vegetation and hydrological characteristics were related to mosquito abundance and community composition. Given the differing competences of each species in pathogen transmission, these results provide valuable information on the transmission potential of mosquito-borne pathogens that will be of great use in public and animal health management by allowing, for instance, the identification of the priority areas for pathogen surveillance and vector control.

Vector-borne diseases are an important public health concern^1^ since both the distribution and incidence of many diseases have increased in recent decades^2^. With over 3,500 species worldwide, mosquitoes are by far the most relevant vectors of pathogens^3^ such as metazoan (e.g. filarial nematodes), protozoan (e.g. malaria parasites) and numerous viruses (e.g. Dengue virus, Rift Valley virus and West Nile virus).

The transmission dynamics of vector-borne pathogens are determined by the interactions between the pathogens, vectors and vertebrate hosts, and are influenced by environmental and socio-ecological drivers^4^. Factors such as vector abundance and species distribution are crucial for determining the distribution and incidence of vector-borne diseases^5^. Vector community composition may also affect pathogen transmission given that not all species are competent vectors for a pathogen^6^ and individual vector species play different roles in the transmission cycles of the pathogen. Some species may facilitate the transmission between reservoir vertebrate hosts, while others may act as bridges between infected competent vertebrates and humans or other susceptible species^7^. Moreover, a published model of pathogen transmission dynamic in relation to host and vector biodiversity concluded that heterogeneity in the susceptibility of the reservoir species could dilute the pathogen transmission, while greater vector species richness may amplify its circulation^8^. In this work, Roche *et al*. suggested, using mathematical models, that both mosquito and bird species diversity are important for the transmission dynamics of vector-borne pathogens (e.g. West Nile virus (WNV))^8^.

Understanding how environmental variables influence the distribution of mosquitoes is a key issue in disease ecology, especially since anthropogenic changes in climate and landscape (global change) are now affecting the distribution and incidence of vector-borne pathogens^9^. It is widely accepted that environmental characteristics affect mosquito distribution^10^,^11^ since each mosquito species has certain habitat requirements, which can vary greatly even between closely related species^12^.

Human activities produce substantial ecological disturbances that affect communities of both vertebrate and invertebrate organisms^13^,^14^, and often lead to an increase in the abundance of a few species and a general loss of biodiversity^15^. Therefore, the increased dominance of a few key species, both vector and/or host species, and the ecological conditions (e.g. anthropized environments) could favour their interspecific contact rates (i.e. increased biting frequency) and pathogen transmission rates^16^. Factors including the simplification of habitat structures^17^ and the alteration of trophic interactions^18^ may govern anthropogenic-mediated loss of biodiversity in urban areas. Changes in resource availability, vegetation coverage, the characteristics of water bodies, and both temperature and rainfall patterns may all have a severe impact on mosquito populations^12^,^19^,^20^ and thus directly and/or indirectly affect their community ecology^14^ and the pathogen transmission risk (reviewed in LaDeau *et al*.^21^,^22^).

Mosquito communities in urban and rural landscapes are generally characterized by a lower mosquito diversity and/or abundance than in natural areas^12^,^23^,^24^. Although marshlands and other temporary flooded areas are generally absent from urban areas, human activity creates artificial habitats such as water deposits, swimming pools, urban sewage systems, gardens, and subterranean and storm water systems that act as alternative breeding sites for mosquitoes. These habitats appear to be suitable for *Anopheles*^25^, *Culex*^26^ and *Aedes* mosquitoes^27^, especially during the dry season when surface water is otherwise scarce. Therefore, the effects of urbanization may vary depending on the mosquito species as some species may be favoured by anthropogenic environmental changes^13^. By contrast, other species may react differently to such change and their abundances may decrease on an urban-to-rural gradient. For instance, *Culex* mosquitoes were more common in urban sites while mosquitoes of the *Mansonia* genus predominated in rural habitats^10^. In addition to these species-specific responses, mosquito community composition and abundance may also play an important role in pathogen transmission^7^,^28^. The varying degrees of implication of each species in pathogen transmission depend on their vectorial competence, blood-feeding behaviour and life history traits. Therefore, it is vital to identify the ecological factors that drive the abundance of mosquito vectors of pathogens and thus ultimately may affect pathogen transmission.

Here, we integrate field data collected at 45 sites along an urbanization gradient to assess the impact of land use on mosquito abundance and community composition (Fig. 1). The study was conducted in areas with differing degrees of anthropization ranging from natural areas with little human-driven landscape transformation, to rural areas characterized by the presence of livestock and anthropized urban areas with dominance of human presence. We expect that results from this study allow to better understand how landscape characteristics are related to the amplification and transmission dynamics of different mosquito-borne pathogens such as heartworms^29^, West Nile and Usutu viruses^30^,^31^, and avian malaria parasites^32^ that are endemic in southwest Spain.

## Results

### Mosquito abundance and species composition

A total of 340 829 female mosquitoes belonging to 13 species and five genera were collected. The most trapped species were *Culex theileri* Theobald (282 891 ind.), *Ochlerotatus caspius* Pallas (21 155), and *Culex pipiens* Linnaeus (19 268), followed by *Culex perexiguus* Theobald (5,939), *Anopheles atroparvus* Van Thiel (5,387), *Culiseta annulata* Schrank (2,514), *Ochlerotatus detritus* Haliday (1,495), *Culex modestus* Ficalbi (1,237) and *Culiseta longiareolata* Marcquart (476). Most *Anopheles atroparvus* were captured at a single study site, the Doñana National Park, where 3,207 individuals were collected. We also captured a number of additional species in very low number: *Anopheles algeriensis* Theobald (41), *Ochlerotatus berlandi* Seguy (22), *Culiseta* s*ubochrea* Edwards (13) and *Urotaenia unguiculata* Edwards (6). Finally, 216 *Anopheles* spp., 167 *Culex* spp. and two *Culiseta* spp. could only be identified to genus level. Mosquitoes not identified to species level were excluded from species richness and diversity index calculations.

The average number of mosquito captured per trap night was 424.33 (range 4.28–5,780.89), the average species richness per locality was 6.75 (range 2–10) and the average mosquito diversity per locality was 0.41 (range 0.06–0.71), see data for each locality in Supplementary Table S1.

### Environmental effects on mosquito community

General Linear Mixed Models (GLMM) indicate that mosquito abundance and species richness were similar in natural and in rural areas but were greater than in urban areas (Table 1). The captures of each of the commonest mosquito species were lower in urban areas than in natural ones. However, the abundance of *Cx. modestus* and *Cx. perexiguus* were similar in rural and both urban and natural areas. A significantly lower abundance of *An. atroparvus, Cx. theileri* and *Oc. caspius* was found in urban than both rural and natural areas (Table 1). The abundance of *Cx. pipiens* was similar in urban and rural areas but lower than in natural areas (Table 1). *Cx. pipiens* was by far the most abundant mosquito species in urban areas, while *Cx. theileri* was the most abundant species in rural areas (Table 1).

Random Forest (RF) results support a close association between landscape and hydrological characteristics, Normalized Difference Vegetation Indices (NDVI), and mosquito community variables (Table 2). The best models for total mosquito abundance and species richness were those that included environmental variables measured at the 1000 m and 250 m radii buffers, respectively. The total abundance of mosquitoes was positively associated with the area occupied by wetlands (Fig. 2a) but negatively associated with both the area of urban land (Fig. 2b) and the human population (Fig. 2c, Table 2). Similarly, mosquito species richness was negatively associated with the area occupied by urban land (Fig. 2d), the human population (Fig. 2e) and the distance to marshlands (Fig. 2f, Table 2). Table 2 summarizes the most important variables explaining the abundance of the commonest mosquito species sampled. *Cx. theileri* was the most abundant species in the studied areas and its abundance was negatively related with the area occupied by urban land (Fig. 2g) but positively related to the area occupied by wetlands (Fig. 2h, Table 2). The abundance of the majority of mosquito species was positively related to summer NDVI. Positive associations were found between the abundance of *An. atroparvus, Cx. modestus* and *Cx. perexiguus, Cx. theileri* and the summer NDVI (Fig. 2i), and the abundance of *Cx. perexiguus* and autumn NDVI (see Supplementary Fig. S1 for further details)*. Cx. modestus* abundance was negatively related to winter NDVI (Supplementary Fig. S1). Additionally, the abundance of *Cx. modestus* and *Oc. caspius* was negatively related to the distance from marshlands (Supplementary Fig. S1). We did not find any association between the diversity of mosquitoes and the abundance of *Cx. pipiens,* and the independent variables included in the RF.

## Discussion

To understand the risk of transmission of vector-borne pathogens it is essential to identify the environmental and biological factors determining the abundance and species composition of mosquito communities^33^. Measuring mosquito abundance in the field is time consuming and financially expensive and so remote sensing variables provide an effective way for identifying which areas are most prone to harbouring significant populations of certain mosquito species^34^. In this study, we found associations between mosquito abundance and richness, and land use in an area of Mediterranean climate, in which several pathogens affecting humans, wildlife and livestock are circulating (e.g. Figuerola *et al*.^30^).

We found strong support for the impact of urbanization on mosquito abundance and community composition and identified some key environmental variables that potentially affect these associations. Mosquito abundance and species richness were higher in natural and rural areas than in urban ones. These results agree with those from previous studies conducted in Europe^24^ and elsewhere^35^ where anthropogenic habitats usually show the lowest abundance of mosquitoes. Natural areas, with freshwater and brackish water wetlands, are more favourable breeding environments, in spite those urban areas may provide suitable habitats for some particular mosquito species (e.g. a wide range of breeding sites in artificial containers and recipients)^9^,^36^. According to our results, mosquito abundance rose as the area occupied by wetlands increased, but fell as the area occupied by urban areas and populated areas increased. Similarly, in South Australia the abundance of different mosquito species increased in areas further from the city centre and closer to saltmarshes^35^. A similar pattern was found for mosquito species richness, which was negatively correlated to factors such as the area occupied by urban land, human density and the distance between urban areas and marshlands. In addition, larvicide treatments with *Bacillus thurigiensis* are carried out in urban areas of Huelva to reduce the nuisance to human populations caused by mosquitoes (S. Ruiz pers. com.) and may have potentially reduced the mosquito populations in some of the studied urban areas. Salt marshes may provide a suitable environment for halophytic species of mosquito^35^,^37^ and the presence of this habitat strongly affected the abundance of species such as *Oc. caspius* in this study. Interestingly, the relationship between mosquito abundance and distance to marshlands was not lineal and had marked thresholds at distances of about 2.5 km for *Cx. modestus* and 10 km for *Oc. caspius*. These differences fit well with the estimated flying distances of mosquitoes, which range between 0.16 km and 1.98 km for *Culex* (e.g. *Cx. pipiens*^38^) and up to 12 km for *Oc. caspius*^39^. According to our results, mosquito abundance and species richness decreased as human population density rose. In this case, the relationship between mosquito abundance and human density had a threshold at areas with approximately 50 habitants/250 m^2^. Previous studies have found strong support for the effect of human population on the transmission of vector-borne pathogens^40^. This may be, at least in part, due to the impact that the reduction in the availability/suitability of breeding areas and the implementation of mosquito control in more densely populated areas have on native mosquito communities. On the other hand, certain invasive species such as *Aedes albopictus* seem to prefer anthropically altered areas to natural landscapes^41^, but fortunately this invasive species, that is colonizing southern and eastern Spain^42^, was not detected during this study.

Close associations were found between the abundance of the commonest mosquito species and environmental characteristics, although these associations varied between species. In particular, we found a general positive relationship between the abundance of the commonest mosquito species and NDVI (an index of photosynthetic activity), especially during the summer season. Higher values of NDVI reflect a higher vegetation covertures and potentially a higher availability of breeding and, especially, resting habitats for mosquitoes and other insect vectors^43^. Previous studies both in USA and Europe have also reported positive associations between NDVI values and mosquito presence, abundance and diversity^44^,^45^,^46^.

*Culex theileri,* the most abundant species trapped in the study area, was extremely abundant in natural and rural areas. Previous studies have found that this species typically feed on blood from livestock and wild and feral mammals^28^. For instance, *Cx. theileri* represent an important vector for *Dirofilaria sp*.^47^ and its great abundance in rural areas may explain the high prevalence of dirofilariasis in the study area^29^. Moreover, the abundance of *Cx. theileri* rose as the area occupied by wetlands increased but fell where urban areas occupied more land. This fact may be due to the breeding requirements of this species. Although it is able to use artificial habitats and heavily polluted water, *Cx. theileri* usually occurs in flooded meadows, stagnant or slowly moving streams, rock pools, swamps and rice paddies, which probably explains its greater abundance in wetland natural areas^12^. Furthermore, *Cx. theileri* is the more abundant mosquito in May–July in the study area^32^,^44^, when the environmental conditions reflected by the summer NDVI index (higher vegetation cover during the summer season) are higher. In addition to the higher abundance of this species close to wetlands due to the suitability of breeding areas, rural and natural areas may also provide a higher abundance of potential hosts for this species.

*Anopheles atroparvus* is the only species of mosquito competent for malaria transmission to humans that is present in Spain^48^. It was the most abundant species in rural areas and, similarly to *Cx. theileri*, this species was positively associated with both the area occupied by wetland habitats and greater summer NDVI. Roiz *et al*.^44^ reported a positive relationship between NDVI and inundation area and *An. atroparvus* abundance in the Doñana National Park. In fact, *An. atroparvus* prefers to breed in not polluted sites, with a slight preference for brackish water. This species is commonly found in canals, river margins and paddy fields^12^, which may explain the higher abundance of this species close to wetlands. Moreover, the abundance of *An. atroparvus* was positively related to summer NDVI and its abundance also peaks in summer^44^.

The abundance of *Cx. modestus, Cx. perexiguus* and *Oc. caspius* was greater in natural than in urban environments. Moreover, *Cx. modestus,* was more abundant both in wetlands and in areas with higher summer NDVI (but lower winter NDVI), and its abundance fell as the distance to marshlands increased. This positive relationship with summer NDVI and negative relationship with winter NDVI probably is due to the preference of this species to breed in rice-fields that remain unvegetated during the winter and had very high vegetation cover during the summer season. This species is mainly found in brackish marshes and flooded paddy fields^12^, which are especially abundant in the study area^49^. According to Ponçon *et al*.^50^, this species prefers shallow sunlit habitats and is frequent in meadows and irrigation channels. Analogous results were also found in marshlands from southern Spain^44^, mostly during summer, which may explain, at least in part, the negative association between its abundance and the distance to marshlands. Thus, these results support the potential role of rice paddies as both major sources of mosquito-induced nuisance and an important factor in disease transmission^44^. Furthermore, the abundance of *Cx. perexiguus*, a key link in the epizootic transmission of WNV to horses in southern Spain^28^, was positively related to zones with greater summer and autumn NDVIs. In our study, *Cx. perexiguus* abundance was negatively related to the area occupied by urban land, explaining the higher abundance of this species in natural than urban and rural areas. *Cx. perexiguus* use to breed in stagnant and ephemeral ponds such as swamps, streams, and paddy fields, usually with emergent vegetation especially during later summer and autumn when the abundance of this species peak^44^ while it is usually less abundant during spring^32^. Also, we found strong negative relationship between the abundance of the salt marsh species *Oc. caspius* and the distance to the marshlands. This result supports the finding of Leisnham *et al*.^37^ who reported the influence of tide heights on the abundance of this species. *Oc. caspius* prefers to breed in natural environments^12^,^24^ and usually use salt marshes as breeding sites and areas for larval development^44^. This fact may also explain the higher abundance of this species found in natural habitats in our study. This species is considered a secondary epizootic vector for different and novel flaviviruses of potential medical concern isolated in Spain^51^. Finally, *Cx. pipiens,* an important vector of a large number of viruses and other pathogens including WNV^52^, was the most abundant of all mosquito species in urban areas where it has been suggested to play a key role in the epizootic transmission of pathogens to humans^53^. This mosquito species is commonly found in urbanized areas^52^,^54^,^55^,^56^ where it uses water bodies like vases in cemeteries, small clay pots or water outlets for oviposition and larvae are frequently found in human-made water bodies^55^. In fact, among the six mosquito species analysed, *Cx. pipiens* was the most abundant in urban areas, although its abundance was significantly higher in natural habitats. In The Netherlands, *Cx. pipiens* was a widespread species being present different habitats including wetlands, agricultural and urban sites^24^. In the study area, natural areas provide more suitable conditions for this species. Unfortunately, we failed to identify any significant relationships with none of the analysed variables and the *Cx. pipiens* abundance. This fact is probably due to the incapacity of the spatial resolution of the cartography used here to identify the relevant variables for the ecology of this species. Additionally, the abundance of this species may be strongly influenced by distribution of human infrastructures (i.e. the design of water outlets in the streets) and activities not measured in this analysis.

In conclusion, in this study we provide strong support for the assertion that the anthropization of the landscape is an important factor determining the abundance and community composition of native mosquito species in an area with a Mediterranean climate. In particular, the mosquito species studied here may be implicated in the transmission of diseases affecting humans, wildlife and livestock. Therefore, results reported here on the effect of environmental factors affecting the abundance of particular mosquito species and, given the differing competence of these species in the transmission of pathogens, may provide valuable information for public health management and mosquito control by allowing the identification of priority areas for pathogen surveillance and/or vector control.

## Materials and Methods

### Study areas

**Figure 1:**
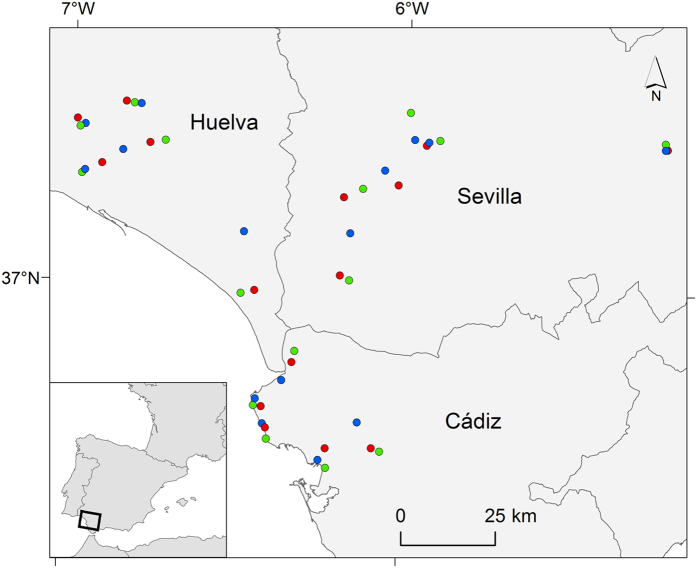
Distribution of the 45 mosquito sampling sites including 15 natural (green), 15 rural (red) and 15 urban (blue) areas. This map was created using ArcGIS v10.2.1 (ESRI, Redland).

**Figure 2:**
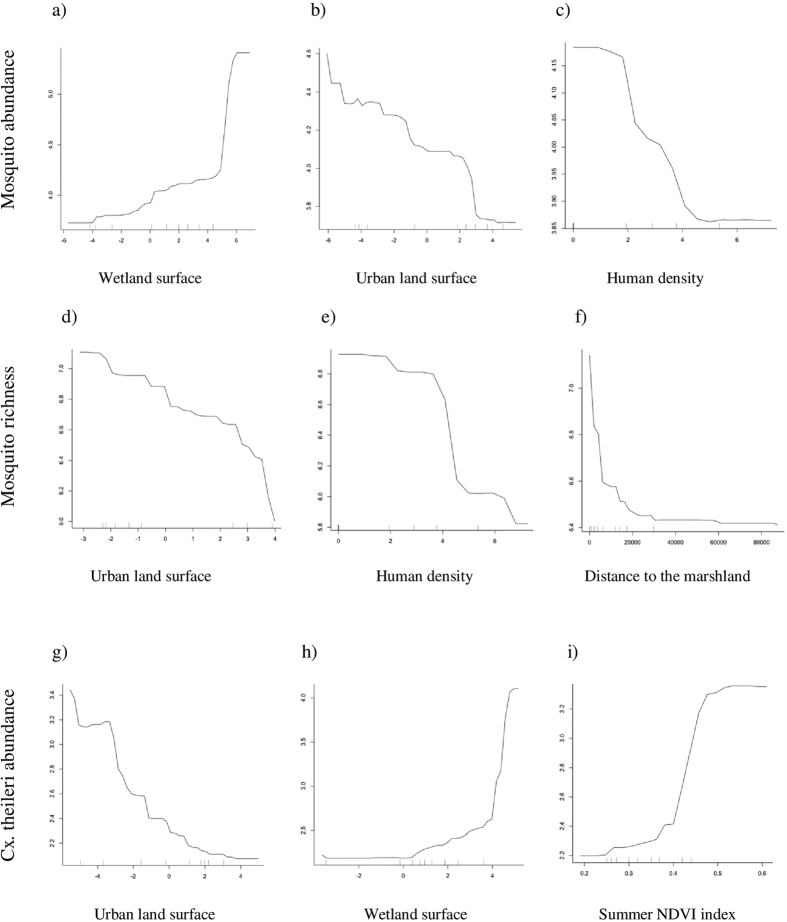
Partial dependence plot for mosquito log-transformed captures and: (**a**) the percentage of land area covered by wetlands (log ratio transformed); (**b**) the percentage of land area covered by urban areas (log ratio transformed); (**c**) human population density (log-transformed). Partial dependence plot for species richness (number of different species) and: (**d**) the percentage of land area covered by urban areas (log ratio transformed); (**e**) human population density (log-transformed); (**f**) the distance to the nearest marshland (m). Partial dependence plot for *Cx. theileri* captures and: (**g**) the percentage of land area covered by urban areas (log ratio transformed); (**h**) the percentage of land area covered by wetlands (log ratio transformed); (**i**) the summer NDVI index. Partial dependence is the dependence of the probability of presence of one predictor variable after averaging out the effects of the other predictor variables in the model.

Our field sites were located in Andalusia (S Spain) (Fig. 1), an area characterized by a Mediterranean climate, with a long dry summer season and greatest precipitation levels in winter. Mosquitoes were captured at 45 different localities in Cádiz, Huelva and Sevilla provinces (15 localities in each province). The sampling sites were grouped in three geographically close localities (named *trios*). Each *trio* included one locality in a natural habitat, one locality in a rural habitat and one locality in an urban habitat according to their environmental characteristics. Urban habitats contained more densely populated areas than the other two habitat types; rural habitats had more heads of livestock than the other two habitat types; natural habitats were selected on the basis of both lower human and livestock densities than in the other two habitat types, and an overall better conserved landscape. The three habitat categories were selected based upon visual inspection of the locations. In order to enhance the statistical power of our analyses, we compared the three habitat categories (natural, rural and urban areas) within the same *trios* to control for any geographically structured factor that may influence mosquito distribution or abundance.

### Mosquito sampling and identification

From April to December 2013, mosquitoes were captured using BG-sentinel traps baited with BG-lure and dry ice as a source of CO_2_, which are known to be very effective for characterizing mosquito diversity and abundance^56^. This period covers the maximum mosquito activity in southern Spain^32^,^44^. Three traps were operated for 24 hours at each sampling site. Overall, 135 traps (3 traps x 45 localities), with a mean distance between traps of 119 m (range 20–636 m), were employed during each mosquito trapping session. Mosquito sampling was conducted once every 45 days and the 5–6 trapping sessions conducted at each site gave an overall trapping effort of 810 trap nights.

Adult mosquitoes were preserved in dry ice and stored frozen until identification. Mosquitoes were separated over a filter paper on a Petri plate on a chill table under a stereomicroscope. Mosquitoes were sorted by gender and date of collection, and then morphologically identified to species level following Becker *et al*.^12^ and Schaffner *et al*.^57^. Specimens belonging to the *univittatus* complex were identified as *Culex perexiguus* based on male genitalia as per Harbach^58^. When several thousands of mosquitoes were captured per trap per night, once we had identified 500 individuals, five groups of 100 individuals were weighted to the nearest 0.001 g, the total number of mosquitoes was estimated from sample weight and the proportion of identified individuals of each species was extrapolated for the rest of the sample.

Analyses were conducted using the following dependent variables calculated for each sampling site: i) total abundance of female mosquitoes, measured as the mean over the whole study period of the mean number of mosquito females of each species captured at each locality on each night, ii) mosquito species richness, measured as the total number of species captured, iii) mosquito diversity estimated using the Simpson index, and iv) the abundance of each of the five most abundant mosquito species – in addition to *Cx. modestus* – that plays an important role in the transmission of pathogens such as WNV^59^.

### Remote sensing variables

Normalized difference vegetation indices (NDVI) were estimated from MODIS sensor images at a spatial resolution of 250 m and a temporal resolution (MOD13Q1 product) of 16 days. Overall, 23 images were obtained to estimate the mean NDVI for each season: winter (images from 03/12/2012 to 18/02/2013), spring (06/03/2013 to 25/05/2013), summer (10/06/2013 to 29/08/2013) and autumn (14/09/2013 to 17/11/2013).

Subsequently, the hydrological and land use information was obtained from cartography accessible at http://www.juntadeandalucia.es/institutodeestadisticaycartografia/DERA/ using ArcGIS v10.2.1 (ESRI, Redland). For the hydrological variables, we measured the distance from each mosquito trap to different water sources (e.g. the distance to the nearest river, marshland patch, stretch of freshwater, the coast and reservoirs), and then calculated the mean distances of the three traps at each of the 45 localities. As well, we calculated the shortest distance to any kind of water source according to the information obtained from the previously described variables. In addition, we quantified land use for different buffers around each mosquito trap using spatial analyses and zonal statistical tools for raster files and the geoprocessing intersect tool for vector variables. The mean value from the three traps at each of the 45 localities was quantified. We considered five different buffers around each trap point with radii of 100, 250, 500, 1000 and 2000 m, which embraced most of the daily flight range of *Culex* mosquitoes (e.g. *Cx. pipiens*^38^). The 33 different land use categories were grouped into four classes: agricultural, forests, wetlands and urban lands (see Supplementary Table S2), and the percentage of the total area occupied by each category was recorded. These land use variables add to 100% and for this reason log-transformed ratios were used for the statistical analyses^60^.

Human density was estimated as the number of people living in a grid of 250 × 250 m developed by the Institute of Statistics and Cartography of Andalusia. The population of each grid cell was taken to be the number of residents registered there on 1 January 2013 according to the local census *Base de Datos Longitudinal de Población de Andalucía.* See Supplementary Table S3 for further details about the variables included in this study.

### Statistical analyses

Firstly, the differences in mosquito abundance, species richness and diversity and the abundance of the six commonest mosquito species between urban, rural and natural areas were tested with General Linear Mixed Models (GLMM). We performed a GLMM for each of the dependent variables, fitted by maximum likelihood functions with Gaussian distribution (package lme4 in R software), including habitat category as a fixed factor and ‘province’ and ‘trio’ as random factors to account for the geographic stratification present in our sampling design.

Secondly, to identify the relationship between environmental variables and mosquito abundance, richness and diversity and the abundance of the six commonest mosquito species we used Random Forest (RF) regression analyses based on 5,000 trees^61^. Random Forest represent iterations of regression trees, whereby both records and predictor variables are randomly permuted to assess the robustness of the derived classifications. This non-parametric algorithm method was used because RF procedures do not require the use of any particular model, which might be difficult to assign given the high number of independent variables. The advantage of RF models is their ability to predict a continuous (as in our case) rather than categorical (presence/absence) variable across a landscape, as well as their ability to assess the relative importance of each variable by predicting a complex model of interactions^62^. No *a priori* assumptions are made about the relationship between predictor and response variables, thereby allowing for the possibility of non-linear relationships.

In the RF analyses, we first included environmental data from the five buffers considered in each model in order to identify the best buffer selected for each variable (see the Supplementary Table S3 for further information about the predictors included in the models). After that, we conducted a second RF analysis including only data from the best-selected buffer using %*IncMSE* (percentage of increase in Mean Square Error) splitting criterion to find the optimal predictors. Finally, we ran a model selection procedure using the VSURF function to identify and plot the set of variables that most influenced our models. *Trio* was included as a stratification factor in all of these analyses. For consistence between the GLMM and the RF analyses, we have used the mean number of mosquito captured at each locality instead of data at the lowest level (considering each mosquito sampling as an independent sample). GLMM’s build using each mosquito trap/night instead of mean values did not differ qualitatively of the results presented here.

The normal distribution of all dependent and independent variables were checked. Moreover, the normal distribution of model residuals was also tested by using *qq plots* in R software. In all cases, residuals followed a normal distribution. Variables were transformed when necessary to reduce the influence of extreme values. Total counts of mosquito, counts of the six mosquito species and human density were log-transformed to normalize its distribution and stabilize the variance and to deal with differences of several orders of magnitude between sampling sites. Statistical analyses were conducted with R version 2.14.2 (R Development Core Team 2005) using the vegan, lme4, car, arm, MuMIn, randomForest and VSURF packages.

### Ethics statement

Mosquito trapping was carried out with all the necessary permits issued by the regional Department of the Environment (Consejería de Medio Ambiente, Junta de Andalucía). Entomological surveys and sampling on private land and in private residential areas were conducted with all the necessary permits and consent, and in the presence of owners. This study did not affect any endangered or protected species.

## Additional Information

**How to cite this article**: Ferraguti, M. *et al*. Effects of landscape anthropization on mosquito community composition and abundance. *Sci. Rep.* **6**, 29002; doi: 10.1038/srep29002 (2016).

## Supplementary Material

Supplementary Information

## Figures and Tables

**Table 1:** Least square means (SE) of mosquito abundance, species richness, diversity and the abundance of the six commonest species of mosquitoes with respect to habitat categories.

Mosquito variable	Urban	Rural	Natural	χ^2^	p
Abundance	2.98 (0.42)^a^	4.27 (0.42)^b^	4.96 (0.41)^b^	19.71	<0.001
Richness	5.46 (0.42)^a^	7.07 (0.42)^b^	7.73 (0.42)^b^	17.88	<0.001
Diversity index	0.34 (0.04)^a^	0.48 (0.04)^a^	0.42 (0.04)^a^	4.84	0.089
*An. atroparvus*	0.23 (0.35)^a^	0.97 (0.35)^b^	0.91 (0.34)^b^	8.02	0.018
*Cx. modestus*	0.16 (0.25)^a^	0.39 (0.25)^ab^	0.79 (0.24)^b^	10.30	0.006
*Cx. perexiguus*	0.20 (0.35)^a^	0.78 (0.35)^ab^	1.05 (0.34)^b^	7.97	0.019
*Cx. pipiens*	2.65 (0.25)^a^	2.54 (0.25)^a^	3.33 (0.25)^b^	7.90	0.019
*Cx. theileri*	0.99 (0.64)^a^	3.20 (0.64)^b^	3.06 (0.62)^b^	24.98	<0.001
*Oc. caspius*	0.51 (0.38)^a^	1.85 (0.38)^b^	2.29 (0.38)^b^	16.63	<0.001

χ^2^ and p values of each GLMM are shown. Values differing significantly according to Tukey test are marked with different letter.

**Table 2:** Results of the random forest analyses on the total mosquito abundance, species richness and the abundance of the five commonest mosquito species in relation to land-use, hydrological and NDVI variables.

Mosquito variable	Buffer	% Var. explained	Most important variables in model
Abundance	1	45.35	(+) Wetlands, (−) Urban land, (−) Human density
Richness	250	32.06	(−) Urban land, (−) Human density, (−) Marshland
*An. atroparvus*	1	41.25	(+) Summer NDVI, (+) Wetlands, (−) Urban land
*Cx. modestus*	100	19.07	(+) Wetlands, (−) Marshland, (+) Summer NDVI, (−) Winter NDVI
*Cx. perexiguus*	1	26.59	(+) Summer NDVI, (+) Autumn NDVI, (−) Urban land
*Cx. theileri*	2	45.55	(−) Urban land, (+) Wetlands, (+) Summer NDVI
*Oc. caspius*	500	45.76	(−) Marshland, (−) Urban land

No significant models were found for mosquito diversity and *Cx. pipiens* abundance. The most important variables from the models are listed in order of importance and the directions of the relationships are shown in brackets.

*Land use variables*: Urban land = % of land covered by urban areas (log ratio transformed). Wetlands = % of land covered by wetlands (log ratio transformed). Human density = people per 250 m^2^ of land area (log-transformed).

*Hydrological variables*: Marshland = distance in meters to any type of salt marsh.

*NDVI variables*: Summer NDVI = mean summer NDVI. Autumn NDVI = mean autumn NDVI.
