# Supplementary material for: Effects of landscape anthropization on mosquito community composition and abundance
Source: Sci Rep. 2016 Jul 4;6:29002. doi: 10.1038/srep29002 (PMC4931447; doi:10.1038/srep29002)
Supplement: Supplementary Information [file srep29002-s1.pdf]

Supplementary Information

Effects of landscape anthropization on mosquito community composition and abundance

Martina Ferraguti[1*], Josué Martínez-de la Puente[1,4], David Roiz[1,2], Santiago Ruiz[3,4], Ramón Soriguer[1,4], Jordi Figuerola[1,4]

[1] Estación Biológica de Doñana (EBD-CSIC), Seville (Spain).

[2] Present address: Infectious Diseases and Vectors: Ecology, Genetics, Evolution and Control. IRD (Institut de Recherche pour le Développement), Montpellier (France).

[3] Diputación de Huelva, Área de Medio Ambiente, Servicio de Control de Mosquitos, Huelva (Spain).

[4] CIBER Epidemiología y Salud Pública (CIBERESP) (Spain).

* Corresponding author
M.F. E-mail: mferraguti@ebd.csic.es Tel.: (+34) 954466700, Fax: (+34) 954621125.

Supplementary Table S1 – Mosquito mean abundance, species richness and diversity for each locality.

Province	Trio	Habitat category	Locality	Abundance	Richness	Diversity
Cádiz	Chipiona	Natural	Centro de Interpretación de la Naturaleza El Camaleón	19.64	6	0.37
Cádiz	Chipiona	Rural	Hípica La Garrocha	13.06	4	0.42
Cádiz	Chipiona	Urban	Colegio Público Lapachar	11.94	4	0.12
Cádiz	Jerez	Natural	Laguna de Medina	35.39	7	0.41
Cádiz	Jerez	Rural	Yeguada Hierro del Bocado	40.06	6	0.51
Cádiz	Jerez	Urban	OCA La Campiña	13.61	6	0.17
Cádiz	Puerto de Santa Maria	Natural	Centro de Interpretación Casa de los Toruños	105.67	9	0.54
Cádiz	Puerto de Santa Maria	Rural	Granja Carretera del Juncal	32.81	8	0.63
Cádiz	Puerto de Santa Maria	Urban	Camping Puerto de Santa María	22.69	7	0.42
Cádiz	Rota	Natural	Punta Candor	40.89	7	0.54
Cádiz	Rota	Rural	Granja-corral	4.89	5	0.61
Cádiz	Rota	Urban	Camping Aguadulce	27.00	6	0.17
Cádiz	Sanlucar Barrameda	Natural	Pinar de la Algaida	1106.33	9	0.54
Cádiz	Sanlucar Barrameda	Rural	Granja en la Algaida	48.83	7	0.63
Cádiz	Sanlucar Barrameda	Urban	Palacio Duquesa Medina Sidonia	10.56	4	0.44
Huelva	Beas	Natural	Dehesa	64.30	10	0.36
Huelva	Beas	Rural	Ganadería Pan y Ajo	9.40	7	0.56
Huelva	Beas	Urban	Restaurante El Olivo	6.53	5	0.35
Huelva	Doñana	Natural	Control acceso	4640.50	8	0.07
Huelva	Doñana	Rural	Palacio de Doñana	5780.89	9	0.10
Huelva	Doñana	Urban	Oficina SEO en El Rocío	43.56	8	0.57
Huelva	Gibraleón	Natural	Alcornocal de San Isidro	203.92	8	0,35
Huelva	Gibraleón	Rural	Granja	35.00	8	0.49
Huelva	Gibraleón	Urban	Casa particular	9.61	5	0.32
Huelva	Huelva	Natural	Centro de Interpretación Calatilla	129.58	8	0.62

Huelva	Huelva	Rural	Sede Los Álamos Diputación de Huelva	106.67	9	0.49
Huelva	Huelva	Urban	IFAPA	15.78	4	0.21
Huelva	San Juan del Puerto	Natural	Humedal Graveras de las Balastreras	103.20	9	0.58
Huelva	San Juan del Puerto	Rural	Club Hipico Dolmen de Soto	27.73	7	0.71
Huelva	San Juan del Puerto	Urban	Casa particular	29.13	4	0.14
Sevilla	Cañada	Natural	Cañada de los Pájaros	488.56	6	0.58
Sevilla	Cañada	Rural	Dehesa de Abajo	293.44	8	0.45
Sevilla	Cañada	Urban	La HAMPA	18.11	8	0.62
Sevilla	Guadaira	Natural	La Loma del Acebuchar	110.94	8	0.52
Sevilla	Guadaira	Rural	Estación anillamiento Río Guadaira	313.56	8	0.44
Sevilla	Guadaira	Urban	Campus Pablo de Olavide	28.78	7	0.38
Sevilla	Lantejuela	Natural	Laguna del Gobierno	74.60	7	0.15
Sevilla	Lantejuela	Rural	Corral	4.28	4	0.47
Sevilla	Lantejuela	Urban	Piscina	15.58	5	0.38
Sevilla	Sevilla	Natural	Parque El Alamillo	18.86	5	0.60
Sevilla	Sevilla	Rural	Escuela de Equitación "Espigares"	91.00	7	0.52
Sevilla	Sevilla	Urban	Casa particular	11.06	2	0.25
Sevilla	Veta la Palma	Natural	Isla de Tarfia (Veta la Palma)	816.33	9	0.11
Sevilla	Veta la Palma	Rural	Cortijo Veta la Palma	3981.69	9	0.14
Sevilla	Veta la Palma	Urban	Bar-Restaurante Estero (Isla Mayor)	89.06	7	0.58

Supplementary Table S2 – Land use cover. The different categories are grouped into the four habitat classes included in the analyses.

**Land use cover category**	**Habitat class**
Paddy fields	Agricultural
Annual crops associated with permanent crops	Agricultural
Fruit trees	Agricultural
Cultivation mosaics	Agricultural
Olive groves	Agricultural
Mainly agricultural land with natural and semi-natural areas	Agricultural
Permanently irrigated lands	Agricultural
Rainfed agricultural land	Agricultural
Vineyards	Agricultural
Coniferous forests	Forest
Broadleaf forests	Forest
Mixed forests	Forest
Forest-to-thicket transition areas	Forest
Agroforestry	Forest
Rubbish dumps and landfills	Urban
Sports and recreational facilities	Urban
Road and railway infrastructures and associated land	Urban
Continuous urban areas	Urban
Discontinuous urban areas	Urban
Mines and quarries	Urban
Construction sites	Urban
Industrial and commercial areas	Urban
Ports and harbours	Urban
Urban parks and other green areas	Urban
Waterways	Wetlands
Estuaries	Wetlands
Wetlands and marshes	Wetlands
Open water bodies	Wetlands
Heathland and mesophilic scrub	Wetlands
Natural grassland	Wetlands
Beaches and sand-dunes	Wetlands
Saltworks	Wetlands
Peat bogs and meadows	Wetlands

Supplementary Table S3 – Predictors included in (A) the GLMM and (B) RF models used in this study. *Landscape variables were calculated per each buffer.

A)	Variable type	Variable name	Description
Factor	Habitat category	Category	Three kind of habitats (natural, rural, urban), along an urbanization gradient.
Dependent variables	Mosquitoes	Abundance	Total average mosquito captures per locality
		Richness	Number of mosquito species per locality
		Diversity	Simpson index per locality
		An. atroparvus	Total average *An. atroparvus* captures per locality
		Cx. modestus	Total average *Cx. modestu*s captures per locality
		Cx. perexiguus	Total average *Cx. perexiguus* captures per locality
		Cx. theileri	Total average *Cx. theileri* captures per locality
		Cx. pipiens	Total average *Cx. pipiens* captures per locality
		Oc. caspius	Total average *Oc. caspius* captures per locality

B)	Variable type	Variable name	Description
Independent variables	NDVI	Spring	Mean spring Normalized Difference Vegetation Index
		Summer	Mean summer Normalized Difference Vegetation Index
		Autumn	Mean autumn Normalized Difference Vegetation Index
		Winter	Mean winter Normalized Difference Vegetation Index
	Hydrology	Water	Distance in meters to any type of water sources
		Coast	Distance in meters to the seacoast
		Rivers	Distance in meters to any river
		Marshland	Distance in meters to any type of saltmarsh
		Freshwater	Distance in meters to any kind of stretch of freshwater
		Water reservoirs	Distance in meters to any kind of reservoirs
	Landscape*	Agricultural fields	% of land covered by crops (log ratio transformed)
		Forest	% of land covered by woods (log ratio transformed)
		Urban land	% of land covered by urban areas (log ratio transformed)
		Wetlands	% of land covered by wetlands (log ratio transformed)
	Human population	Human density	People per 250 m2 of land area (ln transformed)
Dependent variables	Mosquitoes	Abundance	Total average mosquito captures per locality
		Richness	Number of mosquito species per locality
		Diversity	Simpson index per locality
		An. atroparvus	Total average *An. atroparvus* captures per locality
		Cx. modestus	Total average *Cx. modestu*s captures per locality
		Cx. perexiguus	Total average *Cx. perexiguus* captures per locality
		Cx. theileri	Total average *Cx. theileri* captures per locality
		Cx. pipiens	Total average *Cx. pipiens* captures per locality
		Oc. caspius	Total average *Oc. caspius* captures per locality

Supplementary Figure S1 – Partial dependence plot for *An. atroparvus* captures and: a) the summer NDVI; b) the percentage of land area covered by wetlands (log ratio transformed); c) the percentage of land area covered by urban land (log ratio transformed). Partial dependence plot for *Cx. modestus* captures and: d) the percentage of land area covered by wetlands (log ratio transformed); e) distance to marshland (m); f) the summer NDVI; g) the winter NDVI. Partial dependence plot for *Cx. perexiguus* captures and: h) the summer NDVI; i) the autumn NDVI; l) the percentage of land area covered by urban land (log ratio transformed). Partial dependence plot for *Oc. caspius* captures and: m) the distance to marshland (m); n) the percentage of land area covered by urban land log ratio transformed). Partial dependence is the dependence of the probability of presence of one predictor variable after averaging out the effects of the other predictor variables in the model.

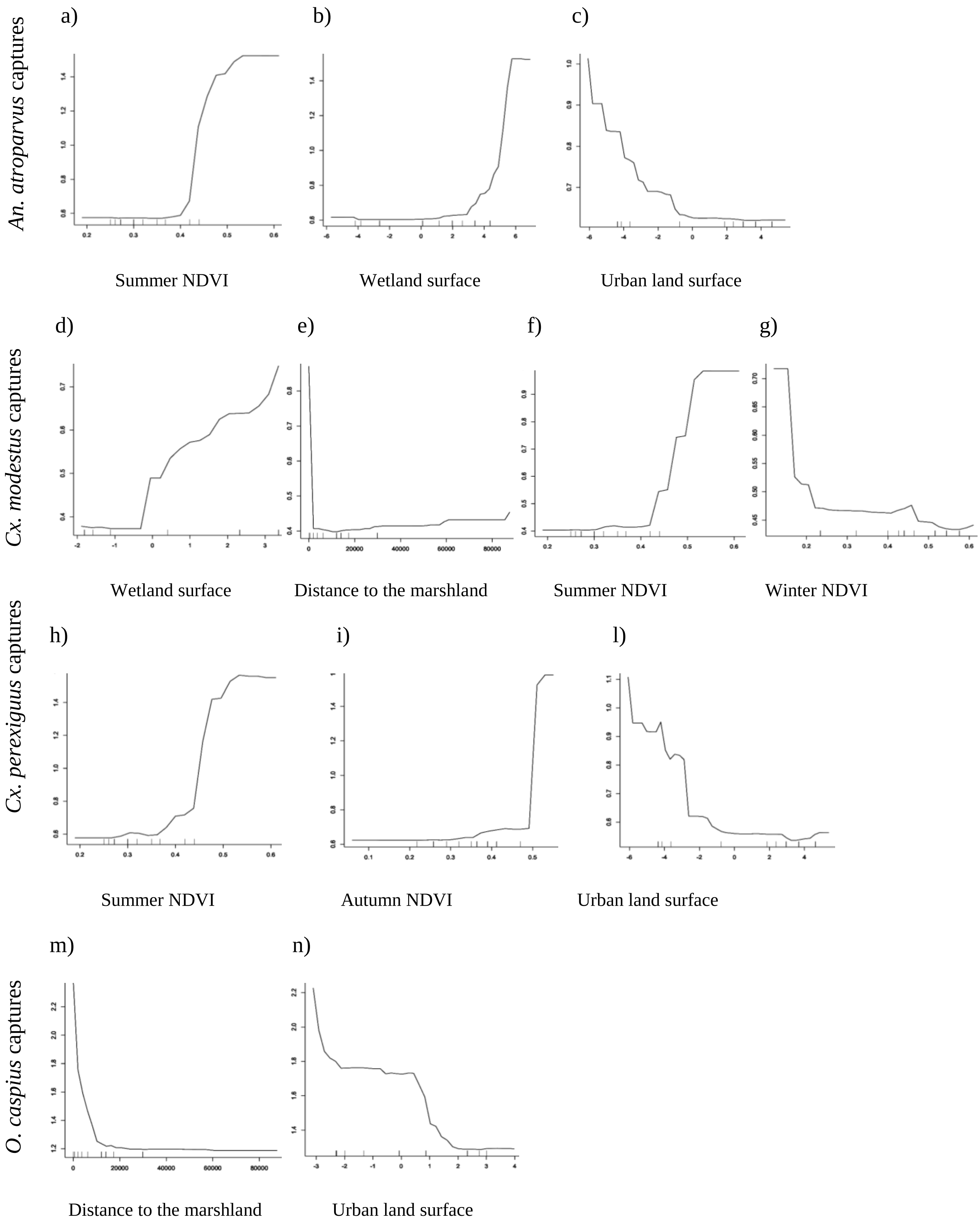
An. atroparvus captures
a)
b)
c)
Summer NDVI
Wetland surface
Urban land surface
Cx. modestus captures
d)
e)
f)
g)
Wetland surface
Distance to the marshland
Summer NDVI
Winter NDVI
Cx. perexiguus captures
h)
i)
l)
Summer NDVI
Autumn NDVI
Urban land surface
O. caspius captures
m)
n)
Distance to the marshland
Urban land surface
